# Coxsackievirus-Induced Myocarditis With Acute Onset of Heart Failure With Pleural Effusion

**DOI:** 10.7759/cureus.55938

**Published:** 2024-03-11

**Authors:** Jaafar A Hamdan, Shaikh Afaq, Akbar Khan, Ritu Shah, Nikolay Mitzov, Maria Castano

**Affiliations:** 1 Internal Medicine, HCA Healthcare/University of South Florida Morsani College of Medicine: HCA Florida Oak Hill Hospital, Brooksville, USA; 2 Graduate Medical Education, HCA Healthcare/University of South Florida Morsani College of Medicine: HCA Florida Oak Hill Hospital, Brooksville, USA

**Keywords:** pericardial effusion, chest pain, shortness of breath, acute heart failure, myocarditis

## Abstract

This is a case of a 45-year-old Caucasian female with coxsackievirus-induced myocarditis. Myocarditis is an inflammation of the heart muscles, which can be difficult to diagnose at times because its symptoms overlap with other cardiovascular diseases. At times, when the patient presents, the full impact of the etiology would have either improved or resolved. In this case, the patient presented with symptoms closely resembling that of acute coronary syndrome but did not fit the typical age category. After lab and imaging workup, the coxsackievirus panel was positive, complicated with a new diagnosis of systolic heart failure with an ejection fraction of 30%-35% along with pericardial effusion.

## Introduction

Myocarditis is an inflammatory cardiac condition of the myocardium, occurring at a rate of about 1-10 cases per 100,000 people per year before the start of the COVID-19 pandemic [1]. Myocarditis ultimately leads to a phenomenon where the cardiac muscles do not adequately contract to perfuse peripheral organs. Myocarditis can ensue from several factors, such as infectious versus non-infectious etiologies. Some of the etiologies include viruses, bacteria (e.g., *Corynebacterium diphtheriae*), parasites (e.g., *Trypanosoma cruzi*), immune system activating factors (e.g., sarcoidosis) or immune-stimulating agents (e.g., vaccines), or exposure to toxins and drugs [1]. In the case of virus-induced myocarditis, few to account for are enteroviruses (e.g., coxsackievirus), vasculotropic viruses (e.g., parvovirus B19), lymphotropic viruses (e.g., cytomegalovirus, Epstein-Barr virus, herpesvirus 6), cardiotoxic viruses (e.g., hepatitis C virus, human immunodeficiency virus, influenza virus), and angiotensin-converting enzyme 2-tropic cardiotoxic viruses (e.g., coronaviruses) [1]. Some of the clinical manifestations that can be found in myocarditis include chest pain, new-onset or worsening heart failure, life-threatening hemodynamic compromise (e.g., fulminant myocarditis with cardiogenic shock and severe impairment of left ventricular function), and lethal arrhythmias or disturbances of cardiac conductive pathways (e.g., ventricular arrhythmias, atrioventricular blocks, sudden cardiac death) [1]. Hence, a thorough evaluation is warranted when a patient presents with a clinical image of heart failure (HF) without any cardiac history, acute onset of chest pain, shortness of breath with physical exertion, peripheral edema with a history suspicious for recent illness, or recent viral illness, as seen in this case. The reason is that patients with reduced left ventricular ejection fraction (LVEF), HF, advanced atrioventricular block, sustained ventricular arrhythmia, or cardiogenic shock are at an increased risk of cardiac transplant or lethal outcomes [1]. Therefore, it is crucial to ensure patients are properly managed and to test for appropriate viral panels given any clinical suspicion based on history and physical examination.

## Case presentation

A 45-year-old female patient with a reported history of chronic obstructive pulmonary disease presented with a chief complaint of shortness of breath along with chest pain. The patient stated that the shortness of breath had been worsening days before admission, particularly with physical exertions, accompanied by a white sputum productive cough. The first episode of chest pain was two weeks prior which had left the patient bedridden for five days. She was unable to move due to severe shortness of breath, fatigue, and chest pain with physical exertion. The pain was diffusely present across the chest, along the nipple line level, described as pressure-like with a severity of 9/10. The patient did not recall how long these episodes lasted. The patient stated that these symptoms began around the same time as she was experiencing upper respiratory symptoms. Initially, the pain had resolved. However, on admission, the patient stated that the chest pain had recurred, present at the same location, non-radiating, sharp in nature, 5/10 in severity, worsening with ambulation, and accompanied by shortness of breath. The patient denied other symptoms including nausea, vomiting, fever, chills, or sweats. The patient’s Thrombolysis in Myocardial Infarction score was 3 and the HEART (history, EKG, age, risk factors, troponin) score was 2. The patient denied any cardiac history. On admission, vitals were a blood pressure of 110/70 mmHg, pulse of 115 beats/minute, respiratory rate of 19 breaths/minute, temperature of 36.5°C, and oxygen saturation of 98% on room air. Physical examination showed the following findings: S1 and S2 appreciated and regular rate and rhythm. A new systolic murmur was appreciated on auscultation. No rubs/gallops were noted. Chest wall tenderness was not present on palpation. Crackles were appreciated on lung auscultation, and bilateral lower extremities 2+ pitting edema was noted. Table 1 presents the abnormal lab findings on the initial encounter, and Table 2 depicts the results of the coxsackievirus panel.

**Table 1:** Patient’s lab abnormalities upon presentation.

Parameter	Values	Reference values
White blood count (10^3^/µL)	12.7	4.0–10.5
Neutrophil (%)	78.8%	34.0–71.7%
Lymphocytes (%)	13.4%	19.3–51.7%
Immature granulocytes (10^3^/µL)	0.05	0.00–0.03
Neutrophils (10^3^/µL)	9.99	1.56–6.13
Monocytes (10^3^/µL)	0.64	0.24–0.63
Chloride (mEq/L)	112	98–107
Anion gap (mmol/L)	3.0	7–16
Glucose (mg/dL)	118	74–106
Aspartate transaminase (U/L)	38	15–37
Albumin (g/dL)	3.3	3.4–5.0
Troponin I (ng/L)	2,736	<54
Pro-B-type natriuretic peptide (pg/mL)	6,582	5–125

**Table 2:** Coxsackievirus panel. IgG: immunoglobulin G; Ab: antibody

Parameter	Values	Reference values
Coxsackie A(7) IgG Ab	1:800 titer	Negative: <1:100 titer
Coxsackie A(9) IgG Ab	1:800 titer	Negative: <1:100 titer
Coxsackie A(16) IgG Ab	1:800 titer	Negative: <1:100 titer
Coxsackie A(24) IgG Ab	1:800 titer	Negative: <1:100 titer
Coxsackie type B(1) Ab	1:16	Negative: <1:8
Coxsackie type B(2) Ab	1:16	Negative: <1:8
Coxsackie type B(3) Ab	1:32	Negative: <1:8
Coxsackie type B(4) Ab	1:16	Negative: <1:8
Coxsackie type B(5) Ab	1:16	Negative: <1:8
Coxsackie type B(6) Ab	1:16	Negative: <1:8

**Figure 1:**
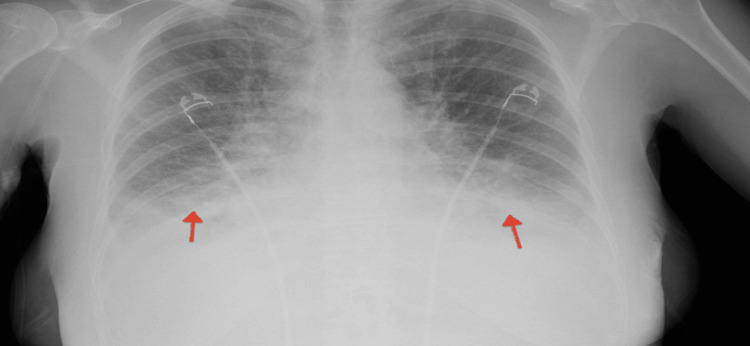
Chest X-ray showing bilateral pleural effusion (red arrows).

EKG showed a sinus tachycardia of 110, low voltage QRS, and no signs of active ischemia/infarction events. On chest X-ray, there was significant bilateral pleural effusion volume overload, as depicted in Figure 1.

**Figure 2:**
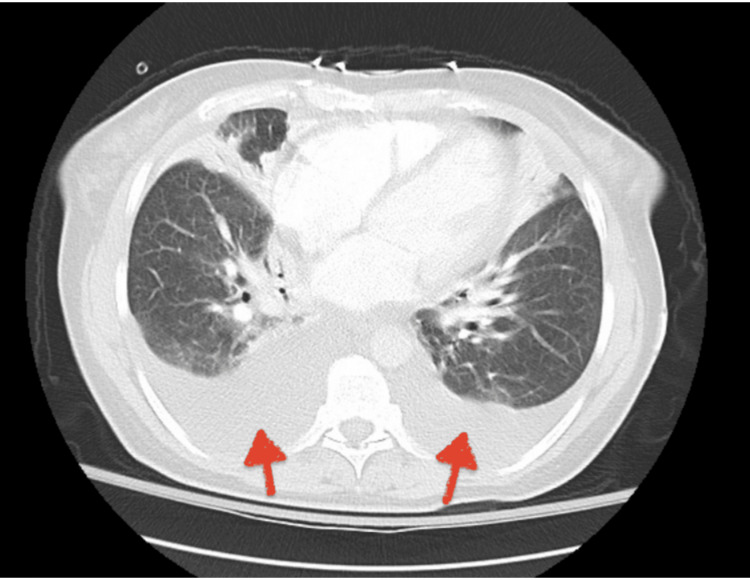
Computed tomography angiography (CTA): Axial view showing significant bilateral pleural effusion (red arrows).

Bilateral pleural effusion was noted on computed tomography angiography (CTA) of the chest (Figure 2).

During the hospital stay, the patient underwent a transthoracic echocardiogram which showed an ejection fraction (EF) of 30%-35% with a moderate to marked reduction in the systolic function. Mitral and tricuspid valves had mild regurgitations and dilation of the left atrium. There was moderate to large-sized pleural effusion (Video 1).

**Video 1:** Transthoracic echocardiogram showing different views with poor contractile mechanics.

**Figure 3:**
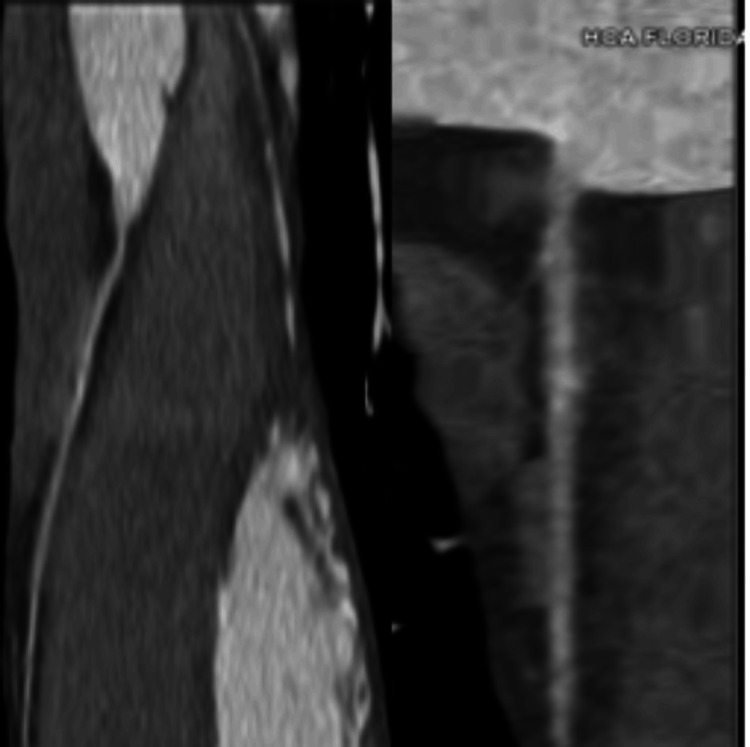
Right coronary artery. Coronary computed tomography angiography shows the right coronary artery being patent without any blockages or stenosis.

**Figure 4:**
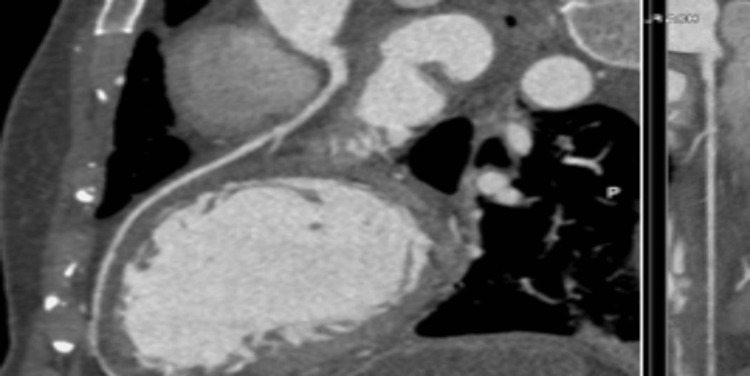
Left anterior descending artery. Coronary computed tomography angiography shows the left anterior descending artery being patent without any blockages or stenosis.

**Figure 5:**
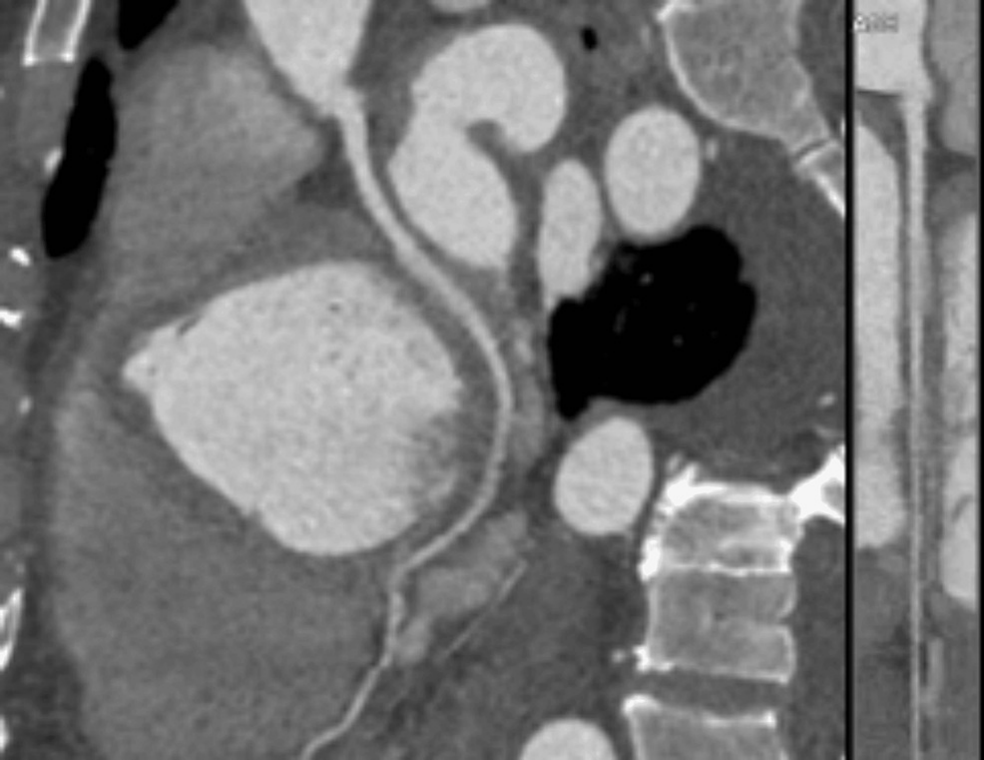
Left circumflex artery. Coronary computed tomography angiography shows the left circumflex artery being patent without any blockages or stenosis.

In addition, the patient underwent a coronary computed tomography angiography (CCTA) which examined different coronary vasculatures (Figures 3-5). It was negative for any coronary pathologies, blockages, or stenosis.

Even though the patient did not undergo an endomyocardial biopsy or cardiovascular magnetic resonance imaging, as it was not available in the hospital, given the patient’s recent upper respiratory symptoms, lack of cardiac history, negative CCTA, positive troponin, N-terminal pro-b-type natriuretic peptide (NT-proBNP), positive coxsackievirus panel, there was strong evidence to conclude a diagnosis of myocarditis.

## Discussion

Myocarditis is an inflammatory cardiac condition of the myocardium, occurring at a rate of about 1-10 cases per 100,000 people per year before the start of the COVID-19 pandemic [1-5]. In the case of COVID-19, a cytokine-releasing event may occur, inducing myocardial inflammation without the presence of a viral genome in myocardial tissue biopsies [5]. A significant factor is cardiac injury due to hypoxemia and coagulopathies which can lead to acute coronary events [5]. Myocarditis can ensue from several factors. Some of the etiologies besides viruses include bacteria, parasites, immune system-activating medical conditions or immune-stimulating agents, or exposure to toxins and drugs [1-4]. In the case of viral-induced myocarditis, one of several to account for is enteroviruses (e.g., coxsackievirus), which is what our patient presented with. Even though the patient did not undergo an endomyocardial biopsy or a cardiovascular magnetic resonance imaging as it was not available in the hospital, given the patient’s recent upper respiratory symptoms, lack of cardiac history, negative CCTA, positive troponin, NT-proBNP, and positive coxsackievirus panel, there is strong evidence that the patient had myocarditis. Moreover, according to the European Society of Cardiology, a non-invasive workup can establish a diagnosis based on clinical/objective findings [1]. In addition to viral etiologies, several classes of pharmacological agents have been associated with myocarditis, such as antipsychotics, immunotherapies, vaccines, and salicylates [1,3]. Clinically, myocarditis can have a variable presentation. Patients can present with chest pain associated with preserved LVEF with no arrhythmias or new onset of HF with reduced LVEF accompanied by a life-threatening hemodynamic compromise [1,2], which is what our patient presented with and on subsequent hospital admission required intensive care unit admission secondary to cardiogenic shock. This is critical to take into consideration that our patient had this initial presentation as this poses a risk for cardiac transplant requirement or in some cases lethal outcomes [1]. As our patient presented with an ejection fraction of 30%, it is critical as heart transplantation or cardiac death occurs mainly in those with LVEF <50%, sustained ventricular arrhythmia, or hemodynamic instability [1].

Diagnosis is ideally based on endomyocardial biopsy, performed mainly in those at a moderate to high risk for myocarditis-related complications requiring inotropic or mechanical circulatory support, ventricular arrhythmia, or Mobitz type II second-degree heart or higher heart block [1,2]. However, as mentioned above, a non-invasive workup can establish the diagnosis based on clinical/objective findings [1]. Some of the ventricular arrhythmia EKG changes that can occur are irregular polymorphic ventricular arrhythmia or regular monomorphic arrhythmia in acute and chronic myocarditis, respectively [1]. When therapy is involved, arrhythmia and HF management must be addressed. For instance, if a patient presents with HF symptoms but is stable, treatments started include angiotensin-converting enzyme inhibitors/angiotensin receptor blockers, beta-blockers, and aldosterone for those with significant low LVEF/persistent symptoms of HF [1]. These treatments along with diuretics were started for the patient upon discharge. Those with hemodynamic instability require inotropic agents; cardiogenic shock refractory to medical management requires mechanical circulatory support with a ventricular assist device or extracorporeal membrane oxygenation (ECMO) may be warranted [1]. A crucial point to keep in mind when evaluating patients with suspected myocarditis is to order proper viral panels when suspecting a case of viral-induced, which was the case of our patient, to avoid misdiagnosis or improper management. Moreover, it is important to obtain an echocardiogram to rule out any pericardial effusion, as seen in our patient, which is a detrimental outcome and can lead to cardiac tamponade if significant. In addition, the patient had detrimental damage to her cardiac muscles which required the placement of an automatic implantable cardioverter defibrillator in a different hospital, and the patient was informed to follow up for continuity of cardiac care as an outpatient.

According to the literature review, other treatments include mechanical circulatory support with a ventricular assist device or ECMO for patients with cardiac shock with severe ventricular dysfunction that is non-responsive to medical treatment. The ultimate treatment is biventricular unloading with adequate systemic and coronary perfusion and venous decongestion which is a bridge to recovery and transplantation or a durable assist device [1]. Furthermore, there have been ongoing clinical trials regarding the usage of high-dose methylprednisolone in those with acute myocarditis associated with HF and/or cardiogenic shock per the MYTHS trial [1].

## Conclusions

Myocarditis is an inflammatory cardiac condition affecting the myocardium, ultimately leading to cardiac muscles not adequately contracting a significant cardiac output to perfuse peripheral organs. Some of the etiologies include viruses, bacteria, parasites, and immune-stimulating agents (e.g., vaccines). In the case of virus-induced myocarditis, it is crucial to keep this as a potential diagnosis when a patient with no cardiac history presents with chest pain, shortness of breath, and HF on a background of recent upper respiratory illnesses. It is important to obtain a thorough history and conduct a physical examination with proper labs and imaging as it can lead to complications such as pericardial effusion and cardiogenic shock, as seen in our patient, which can have lethal outcomes. Hence, proper management from both a medication standpoint and cardiology intervention must be done when deemed necessary.
